# Identifying Potentially Beneficial Genetic Mutations Associated with Monophyletic Selective Sweep and a Proof-of-Concept Study with Viral Genetic Data

**DOI:** 10.1128/mSystems.01151-20

**Published:** 2021-02-23

**Authors:** Yuki Furuse

**Affiliations:** a Institute for Frontier Life and Medical Sciences, Kyoto University, Kyoto, Japan; b Hakubi Center for Advanced Research, Kyoto University, Kyoto, Japan; Princeton University

**Keywords:** evolution, selective sweep, genomes, influenza, ebolavirus, SARS-CoV-2

## Abstract

Genetic mutations play a central role in evolution. For a significantly beneficial mutation, a one-time mutation event suffices for the species to prosper and predominate through the process called “monophyletic selective sweep.” However, existing methods that rely on counting the number of mutation events to detect selection are unable to find such a mutation in selective sweep. We here introduce a method to detect mutations at the single amino acid/nucleotide level that could be responsible for monophyletic selective sweep evolution. The method identifies a genetic signature associated with selective sweep using the population genetic test statistic Tajima’s *D*. We applied the algorithm to ebolavirus, influenza A virus, and severe acute respiratory syndrome coronavirus 2 to identify known biologically significant mutations and unrecognized mutations associated with potential selective sweep. The method can detect beneficial mutations, possibly leading to discovery of previously unknown biological functions and mechanisms related to those mutations.

**IMPORTANCE** In biology, research on evolution is important to understand the significance of genetic mutation. When there is a significantly beneficial mutation, a population of species with the mutation prospers and predominates, in a process called “selective sweep.” However, there are few methods that can find such a mutation causing selective sweep from genetic data. We here introduce a novel method to detect such mutations. Applying the method to the genomes of ebolavirus, influenza viruses, and the novel coronavirus, we detected known biologically significant mutations and identified mutations the importance of which is previously unrecognized. The method can deepen our understanding of molecular and evolutionary biology.

## INTRODUCTION

**FIG 1:**
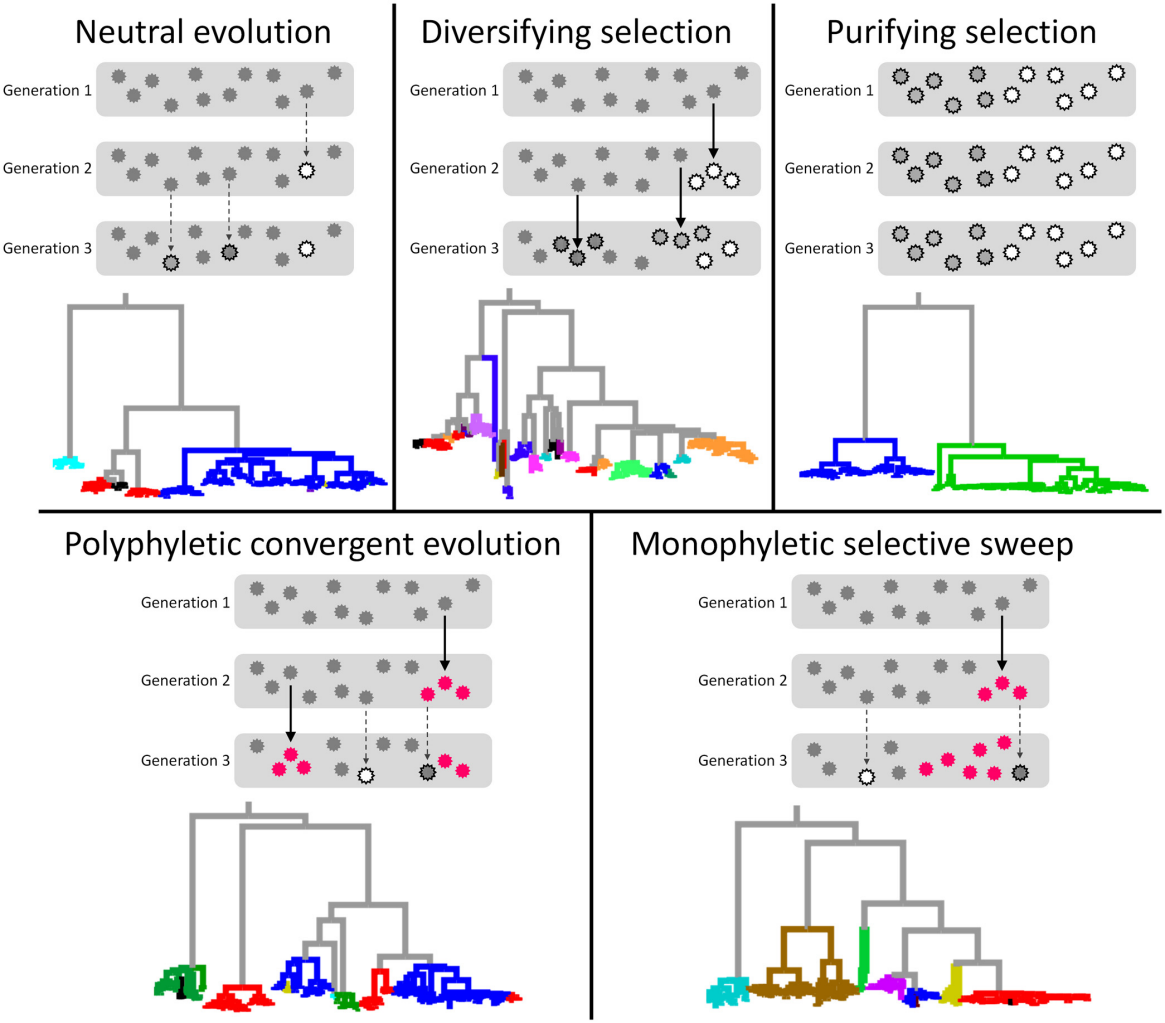
Schematic explanation of different types of selection. Evolutionary pathways and phylogenetic trees are shown for five evolutionary models. The top portion of each panel represents the theoretical concept of each evolutionary model. Gray rectangles indicate a population in the same generation. Viruses with different genetic signatures are depicted as serrated circles with different patterns. As for polyphyletic convergent evolution and monophyletic selective sweep, strains with the beneficial mutation are in red. Beneficial and nonbeneficial mutations are indicated by thick solid and thin dashed arrows, respectively. In this scheme, population size is constant, and increase of fitness is expressed as the increased number of offspring in the next generation. The bottom portion of each panel represents an example of phylogenetic reconstruction from our simulations under each model. Taxa are colored according to the kind of deduced amino acid at a designated position. Strains with the beneficial mutation for polyphyletic convergent evolution and monophyletic selective sweep are in red.

Genetic mutation plays a central role in evolution. Mutations, particularly those in the coding region or functional elements of the genome, can lead to phenotypic change, although most are deleterious and few are neutral or beneficial (1). In this article, we discuss genetic selection at the level of the single amino acid defined by the DNA/RNA codon. The concept of genetic selection at the amino acid level includes diversifying selection, or pressure to keep changing its encoding amino acid (i.e., positive selection in a narrow sense), purifying selection, or pressure not to change its encoding amino acid, and directional evolution, or pressure to possess a specific amino acid. Directional evolution can occur either by polyphyletic occurrences of mutation to a specific amino acid or by population expansion of monophyletic strains with a specific amino acid. We call the first case “polyphyletic convergent evolution” and the second case “monophyletic selective sweep” (Fig. 1).

Rapidly evolving viruses such as influenza A virus are subject to strong evolutionary forces (2). Viruses undergo adaptive genetic mutations within individual hosts and populations of the hosts. Establishing infection in new hosts often requires numerous adaptive changes, such as receptor specificity adjustments (3–5) and overcoming host antiviral defenses (5–7). An example of diversifying selection is the evolution of the hemagglutinin (HA) gene of influenza A virus to evade the host’s immune response, leading to antigenic drift (8–10). Polyphyletic convergent evolution can be observed in the emergence of drug-resistant mutations; for example, the same mutation in the neuraminidase (NA) gene of influenza A virus arose independently in patients treated with oseltamivir (11–13). When a mutation is substantially advantageous for viral replication or transmission, strains with the mutation will reproduce more effectively than other strains, outcompeting those without the mutation (14, 15). This is what we call “monophyletic selective sweep” in this study.

Many methods for detecting signatures of natural selection in genomic data were first applied using viral data because of the rapid evolution of viruses. Calculation of relative ratios of nonsynonymous and synonymous mutations (dN/dS) is a popular way to detect diversifying selection and purifying selection (16, 17). Detection of polyphyletic convergent evolution can be accomplished by studying biased frequencies in protein substitution patterns (18, 19). These methods count the number of mutation events through an evolutionary pathway. Yet a mutation responsible for monophyletic selective sweep occurs only once in an evolutionary pathway. When there is a significantly beneficial mutation, a one-time mutation event must suffice for the viral strain to prosper and dominate an entire population (20). Therefore, the methodology of counting the number of mutation events cannot detect such a mutation leading to monophyletic selective sweep. Methods of detecting selective sweep are available for entire genes or alleles (21), and a few tools to detect a specific region or mutation causing selective sweep are also available (18, 22–25). However, because they do not consider the phylogenetic relationship of strains with a beneficial mutation, they could fail to detect monophyletic selective sweep.

Here, we introduce a method to detect mutations at the single amino acid/nucleotide level that could be responsible for monophyletic selective sweep, Tajima’s *D*-based identification of mutation associated with monophyletic selective sweep (DMAMS). The method detects mutations associated with monophyletic selective sweep with a statistical test using Tajima’s *D*, which is a scaled ratio of two measures of genetic diversity―the mean number of pairwise differences and the number of segregating sites―to test neutral evolution through a site-frequency spectrum (26). A negative Tajima’s *D* value suggests recent selective sweep or population expansion, whereas a positive value suggests balancing selection or sudden population contraction. In this study, we establish our algorithm, DMAMS, which detects a genetic signature (a certain amino acid or nucleotide at a certain position) associated with selective sweep by identifying a monophyletic population with the specific genetic signature that has a large-magnitude negative Tajima’s *D* value. We further demonstrate the application of the algorithm to ebolavirus, influenza A virus, and severe acute respiratory syndrome coronavirus 2 (SARS-CoV-2) to determine whether known biologically significant mutations and unrecognized mutations are associated with potential selective sweep through evolution.

## RESULTS

### Algorithm and concept of DMAMS.

**FIG 2:**
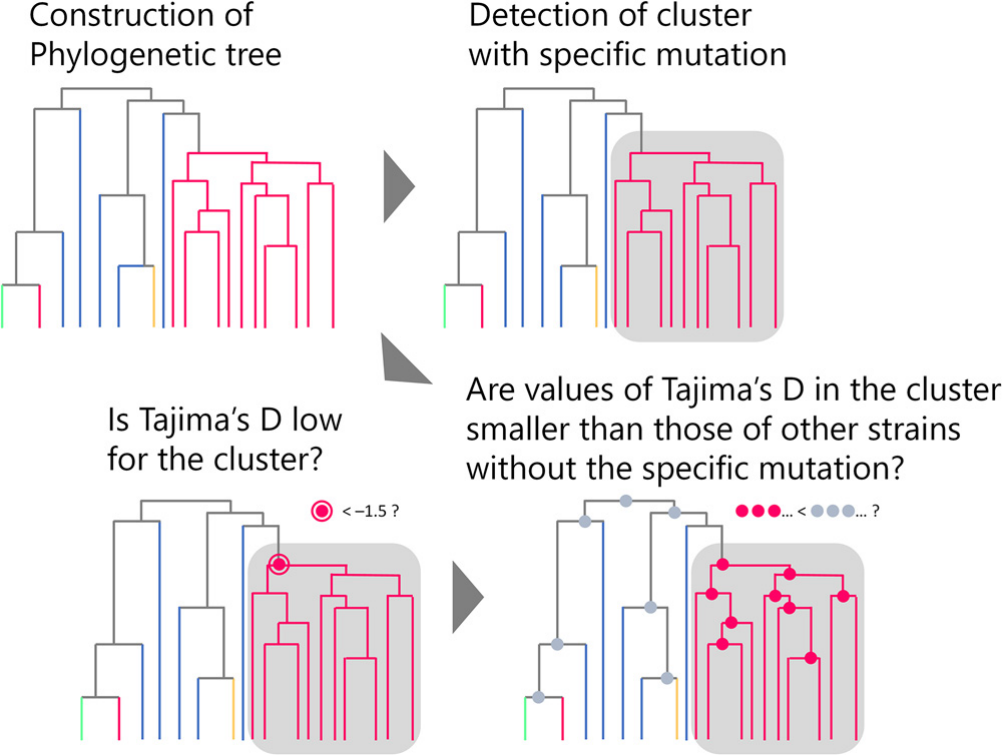
Schematic principle of DMAMS. First, the rooted phylogenetic tree is scanned if there is a cluster with a genetic signature that is rarely shared with strains outside of the cluster. When a cluster with a certain genetic signature is detected, Tajima’s *D* for the cluster is calculated. Tajima’s *D* values for nodes inside the cluster and in the rest of the tree are also computed. There are two criteria to detect a cluster under monophyletic selective sweep: one is an absolute criterion that Tajima’s *D* of the cluster is significantly low, and the other is a relative criterion that Tajima’s *D* values at each node in the cluster are significantly smaller than the values at each node in the rest part of the tree. When both criteria are met, the genetic signature of the monophyletic cluster is considered to have an association with potential selective sweep.

Figure 2 illustrates the DMAMS algorithm. Details of the algorithm are described in Materials and Methods. Briefly, DMAMS determines if there is a monophyletic cluster of strains that share a specific mutation, scanning all 20 amino acids at every position. All nodes in the phylogenetic tree are scanned for each amino acid at each site for clustering. When it finds a cluster of monophyletic strains with a certain amino acid at a certain position that is not shared with strains outside of the monophyletic cluster, Tajima’s *D* for the cluster is computed to check potential selective sweep. Tajima’s *D* values are also computed for internal nodes, comparing the values at nodes in the cluster with the values at other nodes outside the cluster to see the cluster-specific effect.

Population growth and selective sweep are known to have similar effects on Tajima’s *D* (26). The purpose of DMAMS is to detect selective sweep of strains with a certain mutation in a monophyletic subpopulation. Because monophyletic selective sweep is a result of population expansion of a subgroup that consists of monophyletic strains with a certain genetic signature (Fig. 1), differentiation of selective sweep from population expansion is not required in DMAMS. Furthermore, DMAMS will not be affected by changes in an entire population size. When a whole population has expanded and monophyletically clustered strains have shared a certain mutation just by chance, DMAMS will not detect the mutation as associated with selective sweep. That is because DMAMS does not only calculate Tajima’s *D* of a cluster of monophyletic strains with the mutation of interest ensuring potential selective sweep but also compares Tajima’s *D* values at nodes between inside and outside the cluster. That will ensure the cluster-specific effect of population expansion possibly by monophyletic selective sweep.

### Simulation of evolution and performance of DMAMS.

To test the performance of DMAMS, evolution of a viral genome was simulated, and we applied DMAMS to the data generated by the simulations. Details of the simulations are given in Materials and Methods. Phylogenetic trees for virtual viral strains from our simulations reproduced characteristic features of five evolutionary scenarios: neutral evolution, diversifying selection, purifying selection, polyphyletic convergent evolution, and monophyletic selective sweep (Fig. 1). The trees showed unbiased distribution of mutations in a designated site in the neutral evolution model, a wide variety of mutations on the designated site in the diversifying selection model, a conserved polymorphism on the designated site in the purifying selection model, multiple independent occurrences of a designated mutation in the designated site in the polyphyletic convergent evolution model, and rapid expansion of a monophyletic subpopulation of strains with the designated mutation in the designated site in the monophyletic selective sweep model.

**FIG 3:**
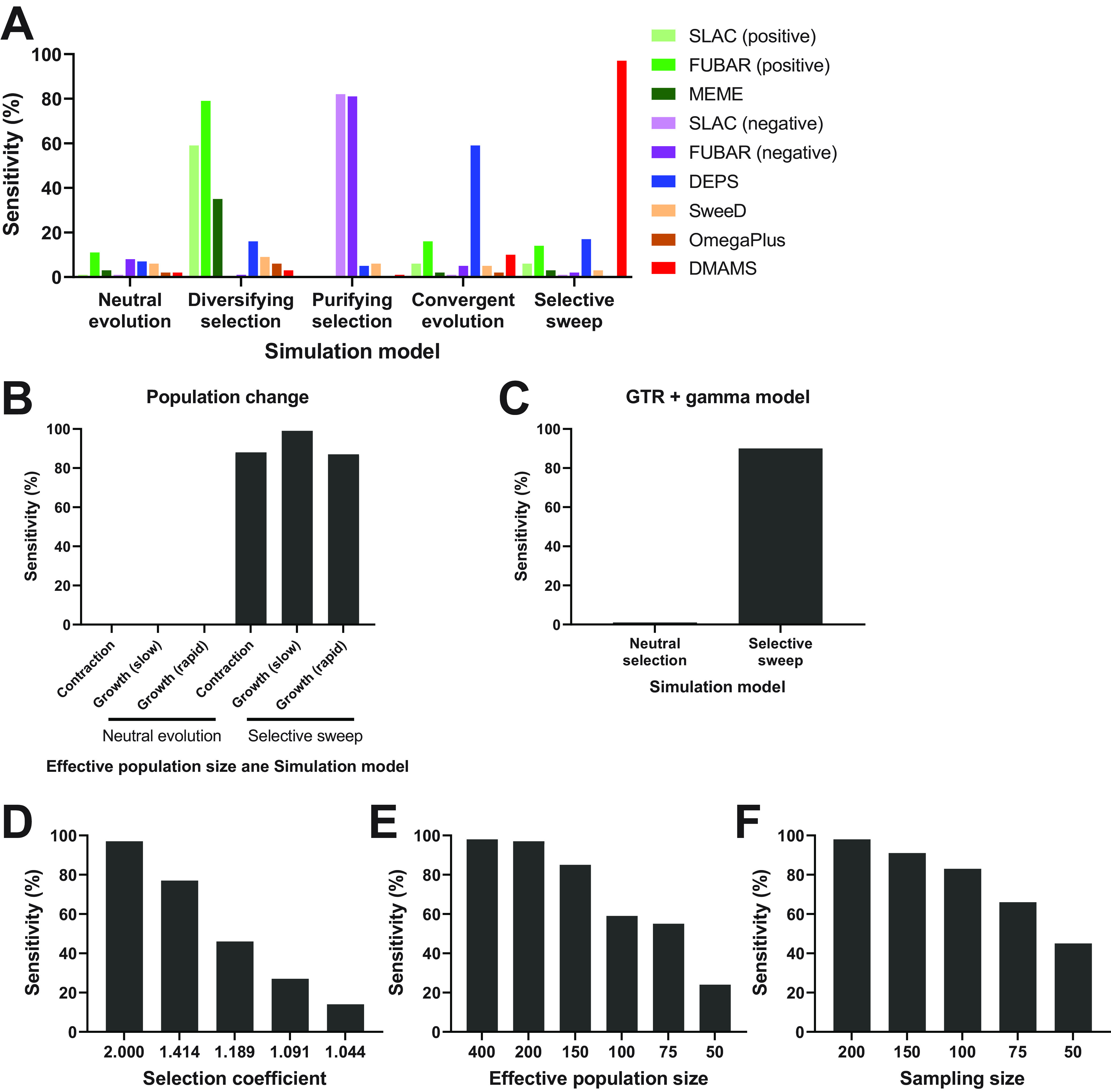
Detection of selection by different methods for different evolutionary scenarios. (A) Proportion of detection of selection at a specific site by various tools for 100 simulations under the neutral evolution, diversifying selection, purifying selection, polyphyletic convergent evolution, and monophyletic selective sweep models. Population size, 200 for all simulations. For the monophyletic selective sweep model, the selection coefficient by a beneficial genetic signature is 2.0. (B) Proportion of detection of selection at a specific site by DMAMS for 100 simulations under the neutral evolution and monophyletic selective sweep models. An entire population size is either decreasing (contraction) or increasing (growth). For the monophyletic selective sweep model, the selection coefficient by a beneficial genetic signature is 2.0. Details of the population change in the simulations are described in Materials and Methods. (C) Proportion of detection of selection at a specific site by DMAMS for 100 simulations under the neutral evolution and monophyletic selective sweep models using the evolutionary model with parameters obtained from the HA gene of influenza A virus. Population size, 200. For the monophyletic selective sweep model, the selection coefficient by a beneficial genetic signature is 2.0. (D) Proportion of detection of selection at a specific site by DMAMS for 100 simulations under the monophyletic selective sweep model according to the selection coefficient by a beneficial genetic signature. Population size, 200. (E) Proportion of detection of selection at a specific site by DMAMS for 100 simulations under the monophyletic selective sweep model according to the effective population size. The selection coefficient by a beneficial genetic signature is 2.0. (F) Proportion of detection of selection at a specific site by DMAMS for 100 simulations under the monophyletic selective sweep model according to the sampling size. Population size, 400. The selection coefficient by a beneficial genetic signature is 2.0.

Algorithms named SLAC, FUBAR, and MEME for analyzing diversifying selection using different assumptions and statistical models (27, 28) detected selection on the designated site in 35% to 79% of the diversifying selection simulations (Fig. 3A). SALC and FUBAR can also be utilized to detect purifying selection. They detected purifying selection on the designated site in 81% to 82% of the purifying selection simulations. Another algorithm for convergent evolution named DEPS (18) detected selection for the designated mutation in the designated site in 59% of the polyphyletic convergent evolution simulations. DMAMS detected the beneficial mutation that had been accumulated polyphyletically by convergent evolution in only 10% of our simulations. Since DMAMS was developed to detect selective sweep of strains with a certain mutation in a monophyletic subpopulation, the sensitivity of DMAMS was not high enough to detect a beneficial mutation under polyphyletic convergent evolution. Those tools, SLAC, FUBAR, MEME and DEPS, detected selection in 3% to 17% of the monophyletic selective sweep simulations. Other tools to detect a region under selective sweep, SweeD and OmegaPlus, also did not detect the beneficial mutation in the monophyletic selective sweep simulations. This is likely because their algorithms are not suitable for detecting selective sweep that has caused a selection advantage in monophyletic strains. Details are presented in Discussion. In contrast, DMAMS detected selective sweep by the designated mutation in the designated site in 97% of the monophyletic selective sweep simulations. The false-positive rate of DMAMS was 2% (positivity in the neutral evolution simulations).

### Sensitivity and specificity of DMAMS in different evolutionary scenarios and population models.

We then tested the performance of DMAMS for a viral population whose effective population size has expanded or contracted because Tajima’s *D* is strongly affected by changes in population size. When a whole viral population has expanded and monophyletically clustered strains in a subpopulation have shared a certain mutation just by chance, DMAMS did not detect the mutation as associated with selective sweep (i.e., few false positives, as shown in the left side of Fig. 3B). Also, when a whole viral population size has contracted but monophyletically clustered strains have predominated because of monophyletic selective sweep by a certain beneficial mutation, DMAMS detected the mutation as associated with selective sweep with high sensitivity (i.e., few false negatives, as shown in the right side of Fig. 3B). The good performance was achieved because DMAMS not only calculates Tajima’s *D* of the cluster of strains with a mutation of interest ensuring selective sweep but also compares Tajima’s *D* values for nodes inside the cluster and the values for nodes in the rest of the tree to see the effect that is specific in the monophyletic cluster of strains with the certain mutation.

We also simulated the evolution of a viral genome assuming a general time reversible (GTR) nucleotide substitution model with gamma distribution of evolutionary rates among sites using parameters obtained from data of the HA gene of human influenza A virus, along with a deleterious effect for most nonsynonymous mutations, to reproduce a more realistic evolution of the viral genome. DMAMS still detected a beneficial mutation as associated with selective sweep in 90% of the monophyletic selective sweep simulations (i.e., true positives) and detected a nonbeneficial mutation in only 1% of the neutral evolution simulations (i.e., false positives) (Fig. 3C).

The high sensitivity in detecting a genetic signature causing monophyletic selective sweep by DMAMS was achieved when the selection coefficient was 200% (i.e., strains with the designated genetic signature were twice as likely to produce offspring in the next round as were strains without the genetic signature) (Fig. 3A to C). The sensitivity of DMAMS depends on the degree-of-fitness increase by the genetic signature (Fig. 3D). The sensitivity of DMAMS also depends on the effective population size and sampling size (Fig. 3E and F). The increase in Tajima’s *D* value is attributed to the low sensitivity of DMAMS in evolutionary scenarios with a small selection coefficient and small effective population size (see Fig. S1A to C in the supplemental material). Changing the parameter setting for “minimum size of cluster to test” in DMAMS might restore sensitivity in some scenarios (Fig. S1D). On the other hand, the specificity of DMAMS was robust over those different evolutionary scenarios and settings in DMAMS (Fig. S2).

10.1128/mSystems.01151-20.1 FIG S1 Distribution of Tajima’s *D* and sensitivity of DMAMS in different settings. (A to C) Frequency distribution of Tajima’s *D* values for clusters with a beneficial genetic signature under the monophyletic selective sweep model (“cluster”) along with Tajima’s *D* values for all strains in the same data sets as a reference (“global”), according to the selection coefficient by a beneficial genetic signature (A), the effective population size (B), and the sampling size (C). (D) Proportion of detection of selection at a specific site by DMAMS for 100 simulations under the monophyletic selective sweep model according to the definition of minimum size of cluster. For “small selection coefficient,” the population size is 200 and the selection coefficient by a beneficial genetic signature is 1.189. For “small population size,” the population size is 100 and the selection coefficient by a beneficial genetic signature is 2.0. For “small sampling size,” the population size is 400, the selection coefficient by a beneficial genetic signature is 2.0, and the sampling size is 100. Download FIG S1, EPS file, 0.7 MB. Copyright © 2021 Furuse. 2021 Furuse https://creativecommons.org/licenses/by/4.0/ This content is distributed under the terms of the Creative Commons Attribution 4.0 International license.

10.1128/mSystems.01151-20.2 FIG S2 Detection of selection in the neutral evolution model. (A to C) Specificity was defined as “1 − proportion of detection of selection at a specific site” by DMAMS for 100 simulations under the neutral evolution model, according to the effective population size (A), the sampling size (B), and the definition of minimum size of cluster (C). Download FIG S2, EPS file, 0.01 MB. Copyright © 2021 Furuse. 2021 Furuse https://creativecommons.org/licenses/by/4.0/ This content is distributed under the terms of the Creative Commons Attribution 4.0 International license.

### Application of DMAMS to viral data.

We applied DMAMS to real viral genetic/genomic data. As described in the introduction, DMAMS was developed to detect not sites with frequent mutations but genetic signatures associated with monophyletic selective sweep. Ebolavirus has been introduced into human populations several times (29), and the largest outbreak occurred in West Africa in 2014 to 2015 (30). A mutation at position 82 of the glycoprotein (GP) gene of the virus was reported under positive selection using the calculation of dN/dS (i.e., diversifying selection) (31). The mutation could affect its infectivity, which may have led to the large outbreak (31, 32). The diversifying selection on the site was detected using not only viral genetic data during the 2014-2015 outbreak but also historical data since 1976 (31). Although repeated jumps of the host species barrier from a natural host to humans made the possibly adaptive mutation at position 82 of the GP gene evident, whether the mutation affected the spread of the disease in 2014 to 2015 remains inconclusive (33). In fact, when we analyzed sequence data collected only during the 2014-2015 outbreak, neither SLAC nor DEPS detected positive selection on the site because of the limited number of mutation events on the site in the single outbreak (Table 1). In contrast, DMAMS identified a monophyletic cluster with the genetic signature 82V in the GP gene (Fig. S3A) and found an association with potential selective sweep. The result provides supporting evidence for the importance of the mutation in the spread of the disease during the outbreak. DMAMS also found other genetic signatures associated with monophyletic selective sweep during the 2014-2015 outbreak (Table 1). Among them, 111C in the nucleoprotein (NP) gene and 759G in the RNA-dependent RNA polymerase encoded in the L gene were reported to affect the replication efficiency shown by molecular experiments (34).

**TABLE 1:** Genetic signature of viral gene and tests for selection^*a*^

Virus	Gene	Time	No. of sequences (after exclusion of identical sequences)	Genetic signature of interest	Characteristics of indicated test								
					SLAC		DEPS	DMAMS					
					Diversifying selection^*b*^	Statistical significance	Convergent evolution with statistical significance	Cluster with genetic signature of interest	Low Tajima's *D* (less than −1.5) for the cluster	Smaller Tajima's *D* for strains in the cluster compared with strains without the signature	Statistical significance	Other genetic signatures associated with the same cluster	Detected genetic signatures associated with potential selective sweep (other than genetic signature of interest and other signatures in the same cluster)
Ebolavirus	Whole genome	2014–2015	718	GP-82V	No	NA	No	Yes	Yes	Yes	Yes	VP35-330L, L-759G	NP-111C, VP35-249L, L-39P, L-1645S
H1N1 influenza A virus	Neuraminidase gene	2007–2008	188	275Y	Yes	No	Yes	Yes	Yes	No	NA	354G	45N, 78E, 82S, 86A, 130R, 188M, 249K, 267I, 287I, 329E, 367L, 393V, 451S, 453T
H3N2 influenza A virus	Neuraminidase gene	Intrahost quasispecies (at the commencement of treatment)	66	222V	No	NA	No	No	NA	NA	NA	None	146K, 219T
		Intrahost quasispecies (2 wks after the treatment)	63		No	NA	No	Yes	Yes	Yes	Yes	242I	None
SARS-CoV-2	Whole genome	January–June 2020	2,309	S-614G	No	NA	No	Yes	Yes	No	NA	None	ORF1ab-265I, ORF3a-57Q, N-203K, N-204R

a Phylogenetic trees for the analyses can be found in Fig. S3 in the supplemental material. NA, not available.

b dN/dS of >1 at the site of interest analyzed using SLAC was regarded as diversifying selection.

10.1128/mSystems.01151-20.3 FIG S3 Phylogenetic trees for viral genome/gene for selection analysis. (A) Phylogenetic tree for the whole genome of ebolavirus with 718 viral strains. Strains with the 82V mutation in the GP gene are shown in red. (B) Phylogenetic tree for the NA gene of H1N1 influenza A virus with 188 viral strains. Strains with the 275Y mutation are shown in red. (C) Phylogenetic tree of the NA gene for quasispecies of H3N2 influenza A virus at the commencement of treatment with 66 viral strains. Strains with the 222V mutation are shown in red. (D) Phylogenetic tree of the NA gene for quasispecies of H3N2 influenza A virus at 2 weeks after the commencement of treatment with 63 viral strains. Strains with the 222V mutation are shown in red. (E) Phylogenetic tree for the whole genome of SARS-CoV-2 with 2,309 viral strains. Strains with the 614G mutation in the S gene are shown in red. All phylogenetic trees were constructed using the maximum-likelihood method. Download FIG S3, TIF file, 0.9 MB. Copyright © 2021 Furuse. 2021 Furuse https://creativecommons.org/licenses/by/4.0/ This content is distributed under the terms of the Creative Commons Attribution 4.0 International license.

We next tested if drug-resistant mutations of influenza A virus were associated with monophyletic selective sweep because of the global transmission and spread of drug-resistant strains among human populations (35–37). Oseltamivir-resistant mutations in the influenza A virus have been reported mainly in patients given the antiviral (12, 38), and calculation of dN/dS using sequence data of the NA gene of influenza A(H1N1)pdm/09 virus revealed diversifying selection on a site responsible for the drug sensitivity (39). However, in the 2007-2008 season, a strain of human H1N1 virus with a specific oseltamivir-resistant mutation, 275Y in the NA gene, emerged, and its monophyletic descendants spread to people, including those without a history of antiviral use (36, 40). We analyzed genetic data of the virus collected in that single season, because the effective population size of influenza A virus fluctuates from season to season (41). We identified both a monophyletic cluster with the genetic signature 275Y and sporadic occurrence of strains with the mutation (Fig. S3B). Whereas DEPS identified a significantly high frequency of 275Y substitutions, DMAMS showed that Tajima’s *D* of the monophyletic cluster of strains with the mutation was not smaller than that of the other strains without the mutation (Table 1). The result suggests that there was no association between the monophyletically clustered drug-resistant strains and selective sweep at a population level, which seems reasonable because the antiviral was not administered to most people for prophylaxis or treatment.

We also utilized DMAMS to investigate intrahost viral evolution (i.e., quasispecies). Rogers et al. reported a case of influenza infection by analyzing the mutational spectrum of an H3N2 influenza A virus population sampled from an immunocompromised patient who had shed virus over a 21-month period (42). The patient was infected with quasispecies of drug-sensitive and -resistant viruses during the course of treatment with neuraminidase inhibitors. Although the study by Rogers et al. simply investigated the time course trend of frequency of drug-resistant mutations, DMAMS can take advantage of their data because they used PacBio single-molecule sequencing, which yielded read lengths long enough to perform phylogenetic analysis. It is important to mention that there must be an expansion of population size in intrahost evolution of the virus. Still, DMAMS is not affected by such changes in an entire population size, as shown in our simulation (Fig. 3B).

Two mutations responsible for resistance against a neuraminidase inhibitor, 119V and 222V encoded in the NA gene, emerged during the treatment. Unfortunately, the number of long reads covering the region including position 119 was not sufficient to perform phylogenetic analysis; we focused on position 222. At the beginning of the appearance of the drug-resistant 222V mutation, strains with the mutations did not form a phylogenetic cluster (Fig. S3C). Two weeks after treatment began, the proportion of strains with the drug-resistant mutations increased (42). DMAMS found that the 222V mutation was associated with a monophyletic cluster under potential selective sweep, while the site showed a dN/dS of <1 (Table 1 and Fig. S3D). The results indicate that the drug exerted evolutionary pressure at an individual host level to induce not diversifying selection but monophyletic selective sweep caused by the mutation. Another genetic signature, 242I, was found in the same cluster under potential selective sweep along with 222V (Table 1). Acquisition of a drug-resistant mutation is known to lead to a reduction in viral growth efficiency (43). The 242I mutation warrants further investigation; it may have the potential to compensate for a decrease in viral growth efficiency by a drug-resistant mutation.

Finally, we applied DMAMS to the data of SARS-CoV-2. SARS-CoV-2 appeared at the end of 2019 and caused a worldwide pandemic in 2020 (44). A recent study reported an increase in frequency of a certain mutation, D614G in the S gene encoding spike protein; the mutation potentially increases the fitness of the virus, as shown by clinical and experimental data (45, 46). Still, the significance of the mutation for public health in the real world is controversial (47–49). Using sequence data of the virus published by July 2020 from all over the world, a phylogenetic tree of the virus showed multiple substitution events between D and G on the site and a monophyletic cluster with the genetic signature 614G (Fig. S3E). The site was not under diversifying selection evaluated by SLAC or under convergent evolution toward G detected by DEPS (Table 1). In addition, DMAMS did not show the 614G-specific population growth of monophyletic strains (Table 1). Actually, not only the monophyletic strains with the genetic signature but also strains outside of the cluster without the genetic signature had large-magnitude negative Tajima’s *D* values, −2.79 and −2.80, respectively. The result reflects a rapid growth in population size of the novel virus by a global pandemic in a naive population. There was no significant difference in Tajima’s *D* values between strains with and without the 614G mutation.

## DISCUSSION

Here, we report an approach to detecting genetic signatures associated with monophyletic selective sweep. Our method simply uses genetic sequence information as data and can identify possibly beneficial mutations even if the mutation event occurred only once throughout an evolutionary pathway. We also showed a proof-of-concept application for viral genetic data.

Previously developed tools to detect selective sweep do not consider monophyletic subpopulations with beneficial genetic signatures (22–25). In addition, they were developed to detect a gene or a subregion of a gene under selective sweep; they could not specifically identify a specific site associated with the selective sweep. Furthermore, most of such tools, including SweeD and OmegaPlus tested in the present study, were built for a diploidy genome (24, 25). They assume accumulation of alleles with characteristic genetic signatures by recombination, and they search the region under selective sweep by checking linkage disequilibrium. Therefore, they are not applicable to our idea of monophyletic selective sweep, that is, the expansion of a monophyletic subpopulation caused by a one-time beneficial mutation. Looking at this from the opposite perspective, DMAMS cannot be applied to data that include sequences generated by recombination. This is because DMAMS depends on reconstruction of the evolutionary path by a phylogenetic tree. The phylogenetic tree using whole sequences cannot reflect the true evolutionary path when recombination has occurred.

Another tool to detect convergent evolution, DEPS (18), was not sensitive enough to detect a mutation under directional evolution that is shared only among monophyletic strains (Fig. 3A). Our algorithm, DMAMS, can identify a mutation associated with monophyletic selective sweep even when the mutation event has occurred only once in an evolutionary pathway. Still, it should be noted that DMAMS is not sensitive enough to detect a beneficial mutation shared among polyphyletic strains (i.e., polyphyletic convergent evolution). Such a mutation is not the target for DMAMS. In other words, DMAMS is not a tool to replace existing tools for detecting (polyphyletic) convergent evolution but is a complementary tool to evaluate the significance of mutations shared only among monophyletic strains.

Because DMAMS utilizes a site-frequency spectrum of genetic sequences by calculating Tajima’s *D*, it can detect genetic signatures not only of deduced amino acids in a coding region but also nucleotides in both coding and noncoding regions. With increasing understanding of the functional roles of genetic sequences themselves, including regulation of DNA/RNA stability, transcription and translation efficiencies, and interaction with other molecules (50–53), methods of identifying genetic signatures responsible for selective sweep at the nucleotide level should be of great interest. Unfortunately, because genetic data of noncoding regions were not abundant compared to those of coding regions, we could not find a good example to show this advantage of our method.

We showed that the sensitivity of DMAMS depends on the magnitude of the selection coefficient given by a beneficial mutation. We defined the selection coefficient in our simulations as the likelihood to produce offspring in the next round of generation (Fig. 3D). In real data of viral evolution, the definition of generation is context dependent, such as “a single round of replication in a cell” or “transmission from one host to another,” and those are difficult to observe. Therefore, magnitudes of selection coefficient are hard to be inferred using real viral genetic data. It is important to note that no detection of mutations associated with selective sweep by DMAMS does not necessarily mean that there is no selective sweep, especially when the degree of fitness by a beneficial mutation is not very high.

Our method is limited by the fact that DMAMS cannot detect causal relationships between genetic signatures and selective sweep but simply detects an association between them. Theoretically, DMAMS cannot distinguish a true beneficial mutation causing monophyletic selective sweep apart from “hitchhiking” mutations that were introduced simultaneously with the beneficial mutation (54, 55). There is also a caveat that the data set for DMAMS should include only strains circulating in the same environment. For example, when DMAMS detects a mutation that was found only in a specific geographical area as a mutation associated with potential selective sweep, it is possible that the expansion of the subpopulation is not caused by selective sweep by the certain mutation but is due to external factors that are different between the two areas, such as size and density of the host population, host genetic factors, and environmental conditions. Mutations identified by DMAMS should be further investigated for possible confounding with other factors.

As we have seen, the emergence of mutation and its fixation process can be observed in real time, particularly with rapidly evolving viruses (2, 56). DMAMS can identify genetic signatures associated with monophyletic selective sweep. However, it cannot prove a causal relationship between mutation and selective sweep. Further studies are required to show the biological significance of identified mutations. Even though DMAMS cannot yield direct evidence of the evolutionary significance of a certain mutation, it is a useful tool, like genome-wide association study, to identify a candidate mutation that is potentially important (57). DMAMS has the potential to reveal previously unrecognized biological functions and mechanisms, thereby deepening our understanding of molecular and evolutionary biology.

## MATERIALS AND METHODS

### The DMAMS algorithm.

Aligned genetic (DNA or RNA) sequence data and their associated rooted phylogenetic trees were used to detect genetic signatures associated with monophyletic selective sweep. Figure 2 illustrates the schematic principle of the Tajima’s *D*-based identification of mutation associated with monophyletic selective sweep (DMAMS) algorithm. First, a rooted phylogenetic tree was scanned for a monophyletic cluster with a genetic signature that is rarely shared with strains outside the cluster. Genetic signature can be either an encoded amino acid or a nucleotide at a certain position. DMAMS scans all 20 amino acids (or 4 nucleotides) at every position of sequence data. In addition, all nodes in the phylogenetic tree are scanned for each amino acid (or each nucleotide) at each site for clustering. The minimum cluster size to test can be determined at the user’s discretion. In the present paper, the parameter was set at 5% for the minimum cluster size for all analyses unless otherwise noted. When the proportion of strains with a genetic signature in a monophyletic subgroup was higher than 90% and the proportion of strains with the genetic signature in the rest of the data set was smaller than 50%, the monophyletic subgroup was regarded as a cluster with the genetic signature. Although ideally 100% of the monophyletically clustered subpopulation shares the same beneficial mutation, there might be further mutations resulting in the loss of the beneficial mutation in some strains in the subpopulation. Therefore, we set a 10% margin. For example, when “the minimum size of cluster to test” is 5%, the minimum percentage required for the detection of a potentially beneficial mutation by DMAMS is (5% × 90% =) 4.5%. We regarded that as small enough to detect selective sweep in its early stage.

When a monophyletic cluster with a certain genetic signature was detected, Tajima’s *D* for the cluster was computed. Tajima’s *D* values were also computed for nodes inside the cluster and for nodes in the rest of the tree, excluding the outgroup used for rooting the phylogenetic tree. The nodes for the calculation of Tajima’s *D* should include at least the minimum size of strains for a cluster. Similar inferential methods to measure fitness (i.e., selection coefficient) of strains in a phylogenetic clade are discussed elsewhere (10, 23, 58, 59). We set two criteria to detect a monophyletic cluster under potential selective sweep. One is an absolute criterion that Tajima’s *D* of the cluster is lower than −1.5 (with approximately 90% confidence [26]), and the other is a relative criterion that Tajima’s *D* values at each node in the cluster are significantly smaller than the values at each node in the rest of the tree. The absolute criterion is to detect potential selective sweep for monophyletically clustered strains with a genetic signature. The relative criterion is to see their cluster-specific selective sweep, excluding the effect of changes in effective population size of an entire population. When both criteria were met, the genetic signature of the cluster was considered to have an association with potential selective sweep. For the relative criterion, comparison of Tajima’s *D* values at nodes in the cluster with the values at nodes outside the cluster was made with the Mann-Whitney U test. A *P* value of less than 0.05 was considered statistically significant after adjustment for false discovery using the Benjamini-Hochberg procedure (60). The algorithm of DMAMS was implemented in a script we developed, which is available at https://github.com/yukifuruse1217/DMAMS.

### Simulation of evolution.

The evolution of viral genes was simulated with a constant population size of 50 to 400 or under an exponential growth or contraction population model. The effective population size increases from 10 to 400 at 1,000 rounds of generation in the slow growth model, it increases from 10 to 400 at 100 rounds of generation in the rapid growth model, and it decreases from 1,000 to 200 at 1,000 rounds of generation in the contraction model. The virtual gene was composed of 3,000 nucleotides, and the whole region of the gene had an open reading frame (i.e., 1,000 deduced amino acids). In a given round, each strain produced a number of offspring to populate the next round depending on the fitness of the strain determined by the selection coefficient. Nucleotide mutation (substitution) occurred at a rate of 0.002 per site per round; the substitution rates among four nucleotides were the same (i.e., Jukes-Cantor model). Five evolutionary models for simulations to change fitness according to mutation at a designated site were included: neutral evolution, diversifying selection, purifying selection, polyphyletic convergent evolution, and monophyletic selective sweep.

In the neutral evolution model, every strain has the same fitness regardless of mutations. In the diversifying selection model, strains increase the fitness when any nonsynonymous mutations occur at a designated position. In the purifying selection model, strains decrease the fitness when any nonsynonymous mutations occur at a designated position. In the polyphyletic convergent evolution model, strains increase the fitness when a designated nonsynonymous mutation occurs at a designated position. In the monophyletic selective sweep model, strains exhibit high fitness throughout generations as long as they possess a designated genetic signature at a designated position. The degree to increase fitness (selection coefficient) by the designated mutation was subject to change to investigate the sensitivity of DMAMS, ranging between 1.044 and 2.0. For example, when the selection coefficient is 2.0, strains with the designated genetic signature are twice as likely to produce offspring in the next round than strains without the genetic signature. Simulations were run 100 times for each setting and stopped at 2,000 to 4,000 rounds of generation for neutral evolution, diversifying selection, and purifying selection models. Simulations were stopped when the proportion of strains with a designated beneficial genetic signature exceeded 30% of the entire population for the polyphyletic convergent evolution and monophyletic selective sweep models.

In addition, simulations that are more realistic in reproducing viral evolution were run. We acquired parameters for viral evolution using data of the HA gene of human influenza A (H3N2) virus between 2009 and 2018, assuming a GTR nucleotide substitution model plus gamma distribution for evolutionary rates among sites. Also, in the simulation, we assumed that nonsynonymous mutations decrease fitness except for a designated beneficial mutation. The all-evolutionary simulations were implemented in a script we developed, which is available at https://github.com/yukifuruse1217/DMAMS.

### Tests for evolutionary selection using simulated data.

Genetic sequences of strains at the final round of simulation were sampled along with the original sequence before introducing any mutation to root the phylogenetic tree. Phylogenetic trees were constructed using a maximum-likelihood method with the Jukes-Cantor model or the GTR plus gamma distribution model using MEGA7 (61). The sequence data and phylogenetic tree were used to test diversifying selection by SLAC (27), FUBAR (62), and MEME (63), to test purifying selection by SLAC and FUBAR, to test convergent evolution by DEPS (18), and to test selective sweep by SweeD3.0 (25), OmegaPlus (24), and DMAMS.

### Viral genetic data.

Genetic sequences of the whole genome of ebolavirus collected in West Africa between 2014 and 2015 and the NA gene of human H1N1 influenza A virus from the Northern Hemisphere in the 2007-2008 season were obtained from GenBank via Virus Variation Resource (https://www.ncbi.nlm.nih.gov/genome/viruses). All genetic sequences of the whole genome of SARS-CoV-2 were obtained from GenBank via SARS-CoV-2 Resources (https://www.ncbi.nlm.nih.gov/sars-cov-2/), accessed on 6 July 2020.

Genetic sequences of the NA gene for quasispecies of human H3N2 influenza A virus in a single patient were obtained from SRA (accession no. PRJNA253584) (42). Reads generated by PacBio were mapped to a reference genome [A(H3N2)/California/7/2004], and mapped reads were trimmed to include genetic signatures of interest (amino acid position 222) so that the final data have the maximized number of sequences.

Sequence data including ambiguous nucleotides were removed for further analyses. Strains with identical nucleotide sequences in the same data set were also excluded for two reasons. (i) Tajima’s *D* is a statistic to indicate change in population size and/or existence of selection. Having identical sequences in a data set will not give any additional information on them. (ii) Because DMAMS calculates Tajima’s *D* values at internal nodes of the phylogenetic tree, it will be impossible to compute the values when a node contains a cluster composed only of identical sequences.

Phylogenetic trees were constructed using a maximum-likelihood method based on a GTR substitution model using MEGA7 (61) for influenza virus and RAxML (64) for ebolavirus and SARS-CoV-2. The trees were rooted using an old strain of the same virus. Sequence data, along with the phylogenetic tree, were tested using SLAC, DEPS, and DMAMS to detect sites under evolutionary selection.

### Data availability.

All genetic data analyzed in the study were acquired from public databases, GenBank and SRA, as described above. The source code for DMAMS and evolutionary simulations developed in the present study can be found at GitHub (https://github.com/yukifuruse1217/DMAMS).
