# Supplementary figures and images for: Identifying Potentially Beneficial Genetic Mutations Associated with Monophyletic Selective Sweep and a Proof-of-Concept Study with Viral Genetic Data
Source: mSystems. 2021 Feb 23;6(1):e01151-20. doi: 10.1128/mSystems.01151-20 (PMC8573955; doi:10.1128/mSystems.01151-20)

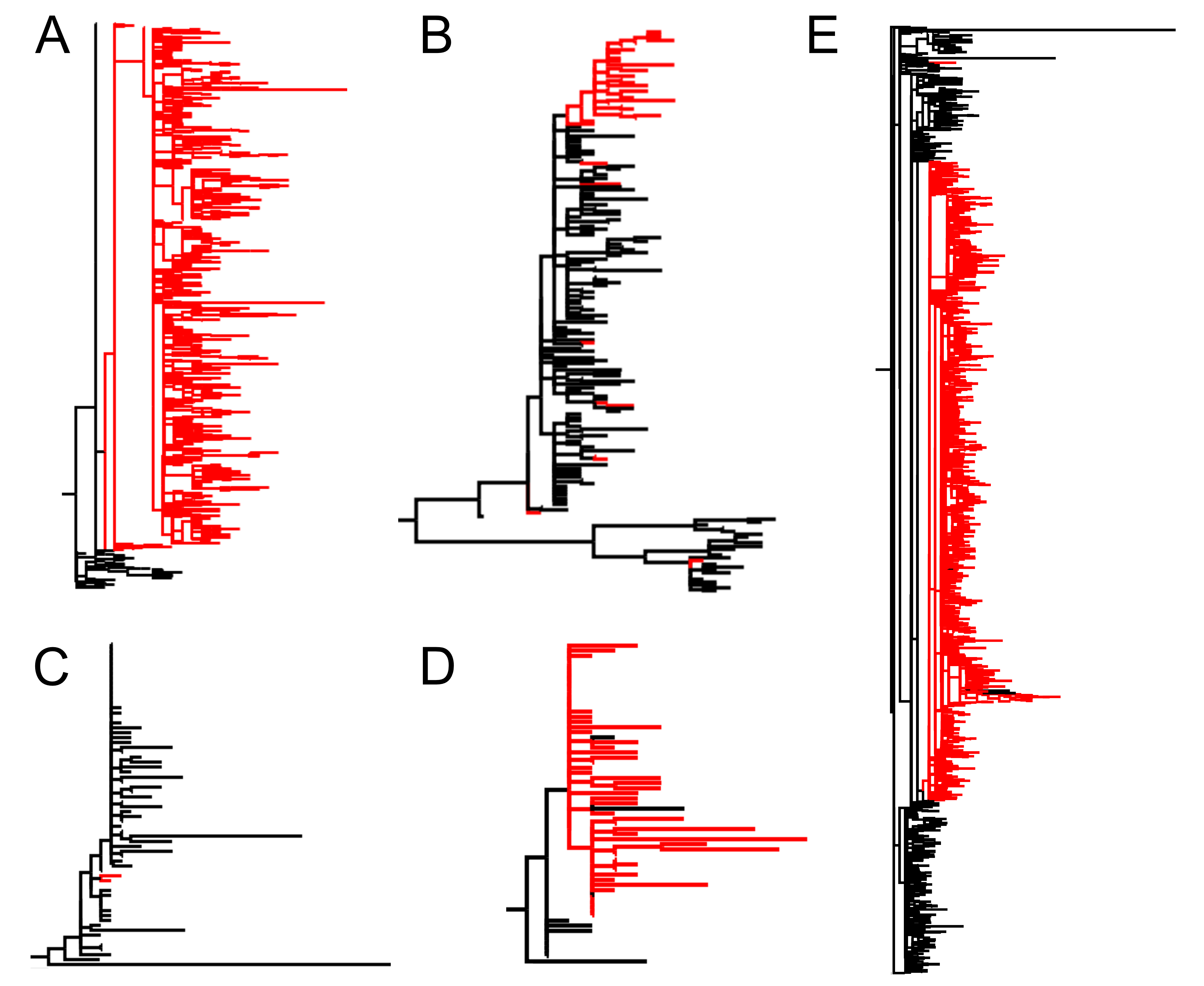

Supplement: FIG S3 [file msystems.01151-20-sf003.tif]
